# Mode and tempo of human hepatitis virus evolution

**DOI:** 10.1016/j.csbj.2019.09.007

**Published:** 2019-10-25

**Authors:** Rachele Cagliani, Diego Forni, Manuela Sironi

**Affiliations:** Bioinformatics, Scientific Institute, IRCCS E. MEDEA, 23842 Bosisio Parini, Lecco, Italy

**Keywords:** Human hepatitis virus, Zoonosis, Molecular dating, Host switch, ORF, open reading frame, NHP, non-human primates, TDRP, time-dependent rate phenomenon, STI, sexually transmitted infection, TMRCA, time to the most recent common ancestor, RdRp, RNA-dependent RNA polymerase

## Abstract

Human viral hepatitis, a major cause of morbidity and mortality worldwide, is caused by highly diverse viruses with different genetic, ecological, and pathogenetic features. Technological advances that allow throughput sequencing of viral genomes, as well as the development of computational tools to analyze such genome data, have largely expanded our knowledge on the host range and evolutionary history of human hepatitis viruses. Thus, with the exclusion of hepatitis D virus, close or distant relatives of these human pathogens were identified in a number of domestic and wild mammals. Also, sequences of human viral strains isolated from different geographic locations and over different time-spans have allowed the application of phylogeographic and molecular dating approaches to large viral phylogenies. In this review, we summarize the most recent insights into our understanding of the evolutionary events and ecological contexts that determined the origin and spread of human hepatitis viruses.

## Graphical abstract

**Figure:**
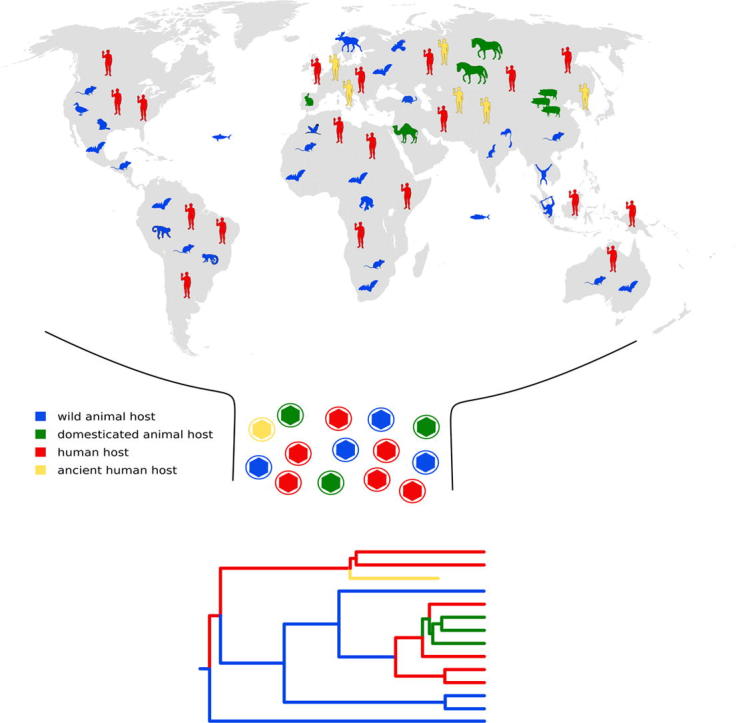

## 1 General introduction

The worldwide burden of viral hepatitis in terms of death and disability is enormous. In 2015, viral hepatitis caused 1.34 million deaths, the majority of which imputable to the effects of chronic HBV (hepatitis B virus) and HCV (hepatitis C virus) infection [1]. An estimated 5% of HBV-infected persons are also co-infected with HDV (hepatitis delta virus), which worsens the clinical outcome compared to HBV monoinfection [1]. Less than 5% of overall hepatitis mortality is caused by HAV (hepatitis A virus) and HEV (hepatitis E virus), that are usually associated with acute, self-limiting disease [1]. However, the case fatality rate of HEV is particularly high in specific groups, notably pregnant women and elderly individuals [2]. Although rare, infection with HAV can also cause acute liver failure and death, and the risk increases with age [3]. Despite the existence of a safe and effective vaccine, HAV remains a common cause of acute viral hepatitis in many regions of the world [4].

Human hepatitis viruses are extremely diverse and consequently belong to different viral families (Table 1). In recent years, the availability of high-throughput technologies has revealed that relatives of human hepatitis viruses can be found in a wide variety of animals. This finding, as well as the increasing availability of the genome sequences of human-infecting viruses sampled across different geographic areas, has largely expanded our knowledge about the genetic diversity and evolutionary origin of these human pathogens. In this Review, we thus focus on the latest insights into the possible events and ecological contexts that determined the origin and spread of human hepatitis viruses. A short presentation of the most widely used approaches to estimate the ages of viral lineages is also provided to contextualize recent research efforts on these viruses.

**Table 1:** Human hepatitis viruses: taxonomic classification and general features.

	Family	Genus	Genome type	Genome length (kb)	Natural host	Course of infection
HAV	*Picornaviridae*	*Hepatovirus*	Linear ssRNA(+)	7.5	Human	Acute
HBV	*Hepadnaviridae*	*Orthohepadnavirus*	Partially circular dsDNA	3.2	Human, non-human primates	Acute/chronic
HCV	*Flaviridae*	*Hepacivirus*	Linear ssRNA(+)	9.6	Human	Acute/chronic
HDV	Unassigned	*Deltavirus*	Circular ssRNA(-)	1.7	Human	Acute/chronic
HEV	*Hepeviridae*	*Orthohepevirus*	Linear ssRNA(+)	7.2	Human, rabbit, pig, wild boar, camel, deer, mongoose, and other mammals	Acute

## 2 Introduction to molecular dating

Molecular dating analyses using virus genetic data can be particularly informative due to the rapid rate of evolution of many viral species. By converting genetic differences among sequences into time units, molecular dating provides information on the timing of viral spread or emergence. Most molecular dating approaches are based on maximum-likelihood or Bayesian phylogenetic frameworks [5], [6], and they usually exploit two strategies: molecular clock calibration using the sampling dates of the viral sequences (tip dating) and/or calibration using information on some internal nodes of the phylogeny. Tip dating is well-suited to study relatively recent events (e.g., epidemics or intra-host evolution), but requires that a temporal signal is present in the dataset (i.e., that the sequences have accumulated a measurable amount of change between sampling times) [6]. Calibration using internal nodes can in principle allow to dig deeper into the past, but requires some *a priori* knowledge about the virus evolutionary history (e.g., host-virus co-evolution, paleontological information). The widespread use of molecular dating has however revealed that the relationship between genetic divergence and time is complex, as evolutionary rates tend to vary with the time frame of measurement. In particular, high evolutionary rates are observed in the short term, whereas low rates are inferred in long time span studies [7], [8], [9]. This pattern was observed for many viral lineages and is sometimes referred to as the time-dependent rate phenomenon (TDRP) [10]. Failure to account for the TDRP can potentially lead to erroneous molecular dating results [10], [11].

The TDRP reflects mutation rate in very short timescales and substitution rate in very long timescales, during which transient deleterious mutations are removed by the action of natural selection, leading to lower rate estimates [12]. Another factor most likely contributing to the TDRP is the saturation of nucleotide substitutions, which is extremely rapid in viral genomes, especially when the polymerase is error-prone [12]. Thus, recent analyses indicated that, for all Baltimore classification groups, viral evolutionary rates tend to decrease continuously with the timescale of measurement [10], [11]. Because the rate of decay is consistent with a power law relationship between substitution rate and sampling timescale, a model using a simple regression was at first proposed to estimate the TDRP effect on viral phylogenies [10]. Very recently, this approach was implemented in a Bayesian statistical framework, in which evolutionary rates can vary among different time epochs [13]. Before the introduction of such an approach, effective attempts to correct for the TDRP were performed by the use of nucleotide substitution models that allow site- and branch-specific variation in selective pressure (selection-informed models). These models, which were applied to analyze the ancient evolution of some viral lineages, at least partially correct for the effects of both purifying selection and substitution saturation in branch length estimation [14], [15], [16].

## 3 Hepatitis A virus

HAV is mainly transmitted via the faecal-oral route through exposure to contaminated food or water, or through direct contact with infected people. HAV is a single-strand, positive RNA virus with a genome of approximately 7.5 kb in length (Table 1). The HAV genome contains a single ORF flanked by a relatively long 5′ UTR and a 3′ UTR. The 5′ UTR harbors an internal ribosome entry site that directs the cap-independent translation of HAV proteins. The ORF encodes a polyprotein processed in 11 mature proteins: 5 structural proteins involved in capsid formation (VP4, VP2, VP3, VP1, and pX, deriving from P1 segment) and 6 non-structural proteins with a role in RNA genome amplification (2B, 2C, 3A, 3B, 3Cpro, and 3Dpol, deriving from P2 and P3 segments) [4], [17].

Based on genomic structure, HAV belongs to the family *Picornaviridae* within the genus *Hepatovirus.* Nevertheless, many characteristics distinguish HAV (and hepatoviruses in general) from other *Picornaviridae* family members. Some peculiar features include the primary tropism for hepatocytes, the ability to shed as nonenveloped virus in feces and as enveloped particles in blood, as well as some genomic features such as low G/C ratio, low CpG levels, and strong codon bias [18], [19].

HAV was identified as the etiologic agent of hepatitis A by Feinstone and colleagues [20] in 1973. Unlike HBV and HCV, which establish chronic infections in humans, HAV infection is usually acute and generates lifelong immunity. This condition is able to determine the disappearance of the virus in small and isolated populations [21], [22] and did not probably favor its maintenance in early human communities. It is thus legitimate to wonder how HAV survived and evolved during early human history, a question that remains presently unanswered.

For a long time, it was thought that hepatoviruses were restricted to humans and non-human primates (NHPs), with genetically distinct variants classified as six main different genotypes [23]: three isolated from humans (HAV, genotype I–III) and subclassified in 6 subgenotypes (IA, IB, IIA, IIB, IIIA, IIIB) and three of simian origin (SHAV, genotype IV–VI). However, despite genetic heterogeneity, HAV viruses belong to a single common serotype.

In recent years, the advent of new sequencing approaches has led to an exponential increase in the identification of new viral species, including highly diverse non-primate hepatoviruses. Several HAV-related viruses were identified in different mammalian orders. In particular, a number of HAV-like viruses were recovered in placental mammals, mainly in bats and rodents, but also in tree shrews, hedgehogs, seals and Chinese woodchucks [24], [25], [26]. Recently, de Oliveira Carneiro and colleagues [27] identified a novel HAV-related virus in *Didelphis aurita*, a Brazilian common opossum, further extending the host range of mammal-infecting hepatoviruses. Moreover, viruses related to mammalian hepatoviruses were detected in reptiles, amphibians, and fish [28]. These advances allowed new insights into the evolutionary history of HAV.

The phylogenetic relationships among hepatovirus that infect small mammals only partially reflects those among their hosts, suggesting multiple, non-recent cross-species and cross-order host switches during hepatovirus evolution [24]. This observation is supported by recombination events observed in hepatoviruses that have been identified in genetically and geographically distant hosts [29]. These cross-species transmission events also involved the opossum hepatovirus, which most likely originated from an ancestral host switch from rodents into marsupials [27]. Conversely, hepatovirus phylogenies suggest no host switch involving a primate donor. This evidence, the absence of recombination events between HAVs and non-primate hepatoviruses, as well as the observation that primate hepatoviruses form, regardless of the genomic region considered, a monophyletic group in the topology of hepatovirus phylogenies, support the hypothesis that humans and NHP have acquired hepatoviruses from other animal reservoirs relatively recently [24], [29]. However, if and when this hypothetical host-jump occurred into primates remains to be clarified.

**Fig. 1:**
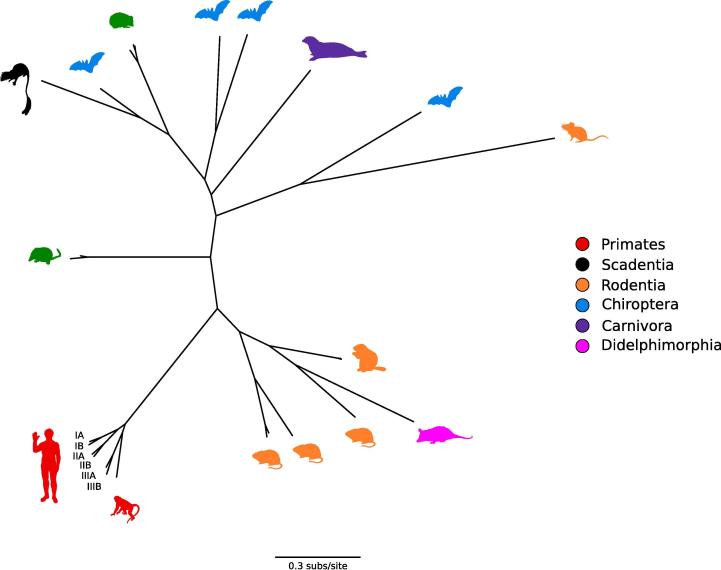
Hepatovirus RdRp domain phylogeny. A maximum-likelihood phylogenetic tree of representative hepatoviruses [24], [27] was generated using PhyML [136]. Hepatovirus host silhouettes are colored according to taxonomic order. The human HAV subgenotypes are also reported.

Phylogenetic and ancestral state reconstruction suggested a likely cricetid rodent origin for primate HAVs and marsupial hepatoviruses, whereas a laurasiatherian host origin was proposed for all mammalian hepatoviruses [24], [27] (Fig. 1). In this scenario, the evolutionary history of hepatoviruses is evocative of that of hantaviruses, as the origin of mammalian hantaviruses is traced back to bats and insectivores [30]. Thus, the supposed origin of hepatoviruses in insectivorous laurasiatherian mammals, as well as the preservation of some structural and functional characteristics similar to present-day insect picorna-like viruses (*Dicistroviridae*) [31] led Drexler and colleagues to hypothesize a more ancient evolutionary origin of HAVs, with an ancestry in a primordial insect-borne virus [24].

In conclusion, HAV emergence in humans likely represents a relatively recent evolutionary event, probably of zoonotic origin. Nonetheless, the ancestor of human hepatoviruses has yet to be identified. The characterization of other hepatoviruses in primates, and mammals in general, will be instrumental to the identification of the HAV ancestors and will clarify the evolutionary history of hepatoviruses.

## 4 Hepatitis B virus

**Fig. 2:**
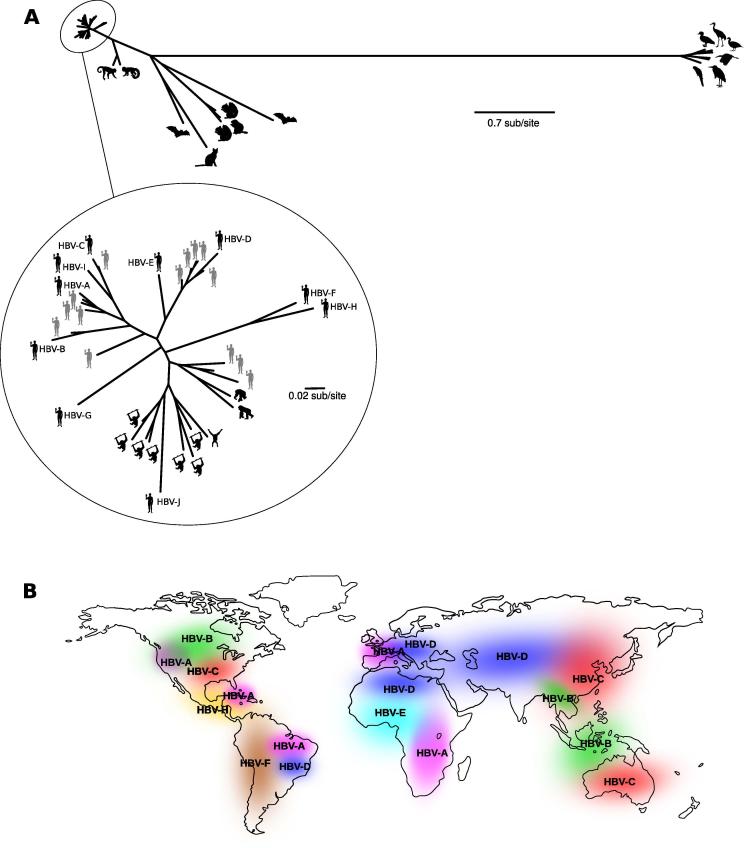
Hepadnavirus diversity and geographic distribution. (A) Maximum-likelihood phylogenetic tree of known hepadnaviruses. Symbols are indicative of viral hosts. The tree of representative hepadnavirus polymerase/reverse-transcriptase (RT) domain was obtained using PhyML [136]. The insert shows the phylogenetic relationship of HBV extant genotypes, HBV ancient strains (in gray), and NHP viruses. (B) Present-day worldwide distribution of the most prevalent HBV genotypes. Geographic distributions derive from Refs. [35], [36], [37], [38], [39], [137].

HBV was the first human hepatitis virus to be isolated and identified in 1970 [32]. HBV transmission varies depending on the prevalence of infection. In areas with a low prevalence (<2%), the most common mode of transmission is through infected blood or high-risk behaviors (e.g. unprotected sex or injecting drug use). In high- and intermediate- prevalence areas, HBV is commonly spread through perinatal and horizontal (especially among children) routes [33]. HBV belongs to the *Hepadnaviridae* family, which comprises two genera: *Orthohepadnavirus* (mammal-infecting viruses) and *Avihepadnavirus* (bird-infecting viruses) (Fig. 2A). Its genome, a partially double-stranded circular DNA of about 3.2 kb (Table 1), is composed of four overlapping frameshifted open reading frames (ORFs) [34]. Viral replication is carried out by a reverse transcriptase with no proofreading ability and considerable variability exists among strains. Thus, at least nine genotypes (A–I) plus a tentative one (J), with a heterogeneous global distribution, have been described to date [35], [36], [37], [38], [39] (Fig. 2B).

Despite its worldwide diffusion and the accumulation of detailed knowledge on the associated pathologies, the origin and evolutionary history of HBV are still debated [34], [40]. Hepadnaviruses were detected in several mammals, including NHPs, rodents and bats, birds, fish, and reptiles [41], [42] (Fig. 2A). Recently, Lauber et al. discovered a family of fish viruses with genomic features similar to those of HBV, dating the origin of the *Hepdnaviridae* family to at least 300 million years ago [43]; this finding, together with the discovery of endogenous hepadnavirus elements integrated in the genome of birds and reptiles [44], [45], [46], [47], suggests a long and complex relationship between this viral family and its hosts.

Concerning HBV, different theories were proposed to explain its origin, but all of them have some sort of limitation. HBV was initially thought to have emerged quite recently in the New World from genotypes F/H infecting Amerindians [48], [49] (Fig. 2A and B). However, the discovery of an ancient strain in a 16th century Asian mummy, as well as the worldwide diffusion of hepadnaviruses in NHPs, questioned this hypothesis [50] (Fig. 2A). An alternative theory suggests that HBV followed the Out-of-Africa migration of modern humans, which occurred approximately 60,000 years ago [51], [52], [53]. In particular, Paraskevis et al. found a good correspondence between the demographic histories of HBV and those of human populations [53]. These authors also showed that the substantial divergence of the F and H genotypes (Fig. 2A), a major evidence in favor of the New World origin hypothesis [49], was probably due to positive selection acting on those branches [54]. Nonetheless, the extensive application of ancient DNA sequencing revealed a more complex scenario. In fact, two European Neolithic HBV genomes did not cluster with any extant human strain in the phylogenetic tree, but did cluster with NHP viruses [55] (Fig. 2A). Other authors [9], who sequenced 12 ancient HBV genomes of different ages (800–4500 years old), obtained a similar result, with the ancient strains clustering with known modern genotypes or forming new clades (Fig. 2A). This implies that some HBV lineages of the past went extinct (Fig. 2A). Moreover, Muhlemann and coworkers showed that the geographic distribution of ancient samples does not match the modern genotype distribution [9]. They thus suggested that early evolutionary scenarios can be concealed and overwritten by more recent migratory events [9].

A third hypothesis for the origin of HBV posits that hepadnaviruses co-speciated with their primate hosts in the New World and in the Old World. Thus, multiple zoonotic transmissions would have originated HBV genotypes found in humans [56]. This scenario is supported by the diffusion of hepadnaviruses in diverse primate species and by the inferred divergence time of the *Orthohepadnavirus* and *Avihepadnavirus* genera, that is very similar to that of their host classes [56]. However, the recent identification of a novel hepadnavirus in capuchin monkeys confirmed that New World monkeys are infected by viruses that are very distantly related to HBV (Fig. 2A), indicating that they do not represent the direct ancestors of genotypes H and F [41]. Instead, evolutionary analyses with human and NHP viral strains placed the origin of HBV ancestors in Hominoid Old World primates, preceding the formation of the human lineage [41].

In summary, although considerable progress was achieved in recent years, a high level of uncertainty concerning the ultimate origin of HBV still exists. The particular organization of the viral genome (i.e. overlapping reading frames in a short genome) limits the variability of most of nucleotide positions (i.e. to avoid the introduction of nonsynonymous mutations) and results in a relatively slow mutation rate. This characteristic, along with the action of natural selection on particular genotypes [54], as well as the adaptation to different human populations [34], contributes to HBV variability and complicates inferences about its origin. Finally, different studies [9], [50], [57] have shown that, as for other viruses (see Section 2), HBV substitution rates are affected by viral sampling time frames. Indeed, the evolutionary rates generated using information from ancient HBV genomes were shown to fit well with the TDRP regression line calculated for Baltimore Group VI and VII viruses (i.e., reverse-transcribing viruses) [11]. Crucially, these results indicate that, whereas tip calibration approaches have demonstrated to be useful in the reconstruction of recent epidemiological events [58], [59], [60], limiting analysis to extant strains for the reconstruction of ancient HBV evolution can be misleading [9] and that approaches that correct for the TDRP should be envisaged.

## 5 Hepatitis C virus

HCV is an enveloped virus belonging to the *Flaviviridae* family (genus *Hepacivirus*). In analogy to other members of the family, HCV has a 9.6 kb positive-stranded linear RNA genome. The virus encodes a single polyprotein that is processed by cellular and viral proteases to yield at least 10 mature products.

HCV was identified in 1989 by Houghton and colleagues as a cause of non-A and non-B hepatitis [61]. If left untreated, HCV can persist lifelong in humans, often resulting in cirrhosis and hepatocellular carcinoma.

Presently, the HCV worldwide seroprevalence is estimated to be ∼1% [1], with about 71 million persons living with chronic infection [1]. The HCV epidemic apparently started recently, in the 1930s–1940s, as a consequence of practices that determined parenteral or percutaneous exposure (e.g., blood transfusion, vaccination campaigns, and intravenous drug injection) [62], [63], [64]. For instance, one of the most affected countries is Egypt, where the virus was most likely disseminated through nationwide vaccination programs or contaminated blood-derived products [65]. In fact, sexual or vertical transmission of HCV are relatively rare, and the overwhelming majority of infections occur via the parenteral/percutaneous route. Thus, due to historical reasons and to the transmission pattern, a small number of so-called “epidemic” HCV subtypes (1a, 1b, 3a, and 2a) account for most infections worldwide [64], [66].

**Fig. 3:**
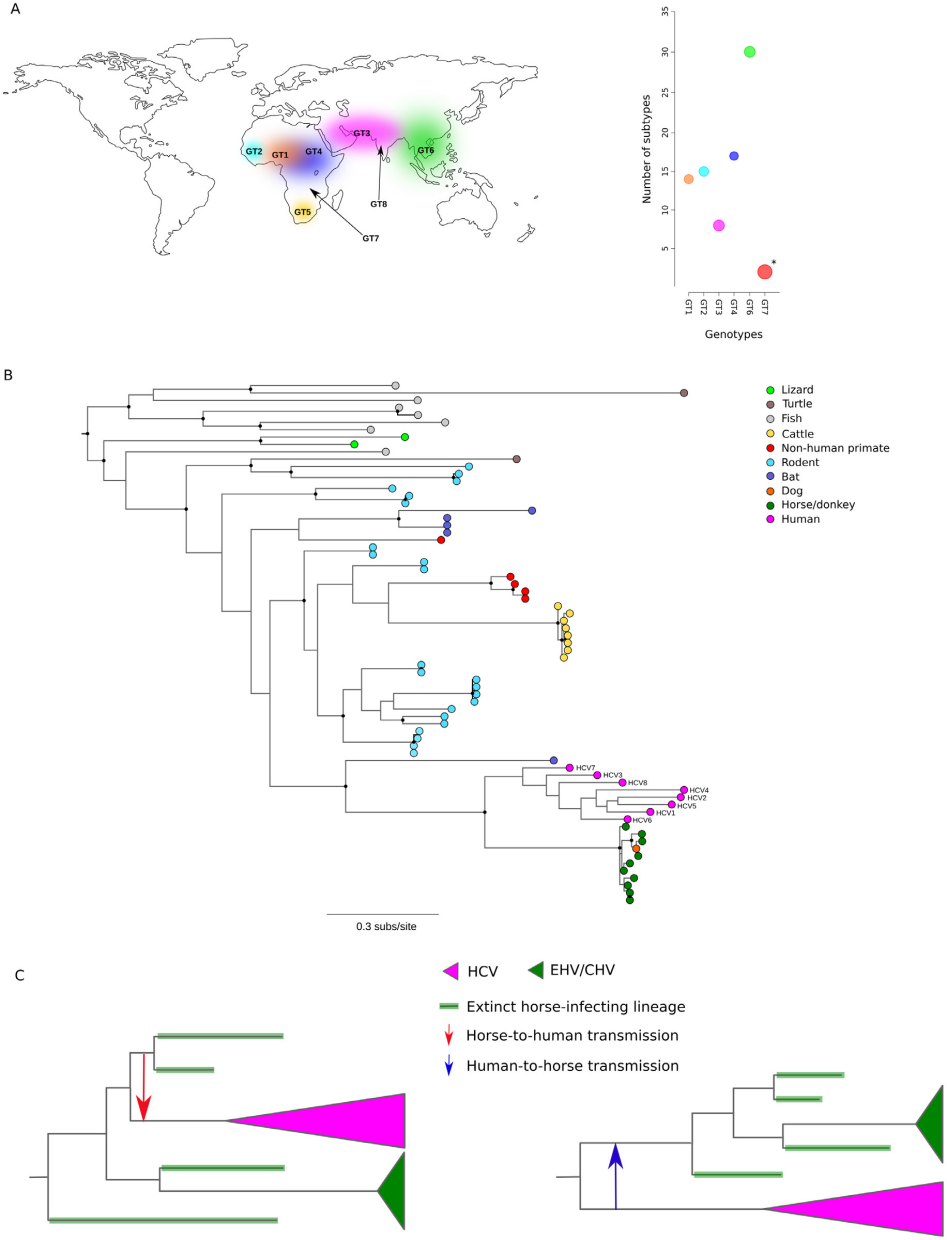
HCV genetic diversity and relationships with other hepaciviruses. (A) Worldmap showing the areas where endemic transmission of different genotypes was reported (data from Ref. [138]). Graphical representation of genotype (GT) diversity. The number of distinct recognized subtypes is represented on the Y axis. Circle size is proportional to the average pairwise distance between subtypes. Genotype 7 is marked with an asterisk as only two subtypes are known. (B) Phylogenetic tree of the RdRp domain of known hepaciviruses. The tree was obtained using RaxML with 1000 bootstrap replicates [139]. Nodes with support equal to or higher than 0.7 are marked with a black dot. (C) Hypothetical viral phylogenies that illustrate the effect of viral lineage extinction on the evolutionary inference about the origin of HCV and EHV/CHV. In the left panel, a horse-to human transmission event is hypothesized, with the following extinction of the transmitted EHV lineage. In the right panel, a reverse zoonosis introduces a HCV-like virus in horse populations; the following extinction of several EHV lineages accounts for the low genetic diversity of extant strains.

HCV is, however, genetically heterogeneous and the epidemic subtypes represent a minor fraction of viral diversity. Eight major HCV genotypes (HCV-1 to -8) have been described, and these are further divided into at least 90 subtypes (https://talk.ictvonline.org/ictv_wikis/flaviviridae/w/sg_flavi/56/hcv-classification) (Fig. 3A). Several of these genotypes and subtypes were identified and classified only recently [67], [68], suggesting that a considerable proportion of HCV diversity remains undescribed. Moreover, a number of natural inter-genotype recombinants were reported (https://talk.ictvonline.org/ictv_wikis/flaviviridae/w/sg_flavi/38/table-4-recombinant-rf-hcv-genomes).

HCV genotypes display antigenic variability and viral genetic diversity is geographically structured: in sub-Saharan Africa and South-East Asia highly divergent subtypes of the same genotype dominate transmissions across contiguous areas (Fig. 3A). These subtypes are referred to as “endemic” [62], [64] and their presence is consistent with a long-standing association of HCV with populations living in these regions. Because parenteral exposure became common only in the relatively recent past, several hypotheses were formulated to account for the maintenance of endemic HCV transmission. Some authors proposed that traditional practices such as circumcision, tattooing, piercing or acupuncture facilitated and maintained HCV infection among human populations [64], [69]. Others indicated that sexually transmitted infections (STIs) that disrupt mucosal integrity are responsible for increased sexual HCV transmission [70]. This was indeed shown to be the case in modern high-risk populations [71] and STIs have probably been common throughout human history [72]. An alternative scenario is that the bite of arthropods, especially those taking large blood meals, can mechanically transmit HCV, possibly from other animal reservoirs such as horses [73], [74]. HCV infection is in fact restricted to our species but, thanks to extensive field work, a number of hepaciviruses have been described in domestic and wild mammals, as well as in reptiles and fish [28], [75], [76], [77], [78], [79], [80], [81], [82], [83], [84] (Fig. 3B). At present, the largest diversity of hepacivirus species seems to be hosted by rodents and bats (Fig. 3B). Instead, as previously noted [75], the lowest genetic diversity is observed for hepaciviruses that infect cattle and horses (Fig. 3B), suggesting that husbandry practices may have resulted in the artificial selection of specific viral strains or facilitated recent viral transmission from some other animal source (e.g., commensal rodents). Non-human primates also host hepaciviruses, but these are highly divergent from HCV. Overall, the phylogenetic relationships among hepaciviruses poorly mirror those among their hosts (Fig. 3B), suggesting several cross-species and cross-order host switches during viral evolution. Up to now, the closest relatives of HCV were identified in horses/donkeys (equine hepacivirus, EHV) and dogs (canine hepacivirus, CHV) (Fig. 3B). Because CHV is less genetically diverse than EHV, the canine virus possibility originated as a recent cross-species transmission from horses [85], hinting at the ability of hepacivirus to shift among genetically distant hosts. Despite these advances, the events that led to the origin of HCV are still unknown. Taking as a fact the relatedness of the human virus to EHV/CHV, possible scenarios include that: i) HCV originated from a cross-species transmission of EHV, ii) that EHV was transmitted to horses by humans infected with HCV, which leaves the question on HCV origin open; iii) that HCV and EHV originated from the cross-species transmissions of the same (or similar) virus, with subsequent host adaptation and divergence. If this were the case, multiple cross-species transmission events may have originated distinct HCV genotypes [85].

Teasing apart these possibilities clearly requires understanding of the timing and circumstances of HCV (and EHV) evolution. Up to date, no archaeological sample carrying traces of HCV or EHV has been described and the oldest HCV sequence dates to 1953 [86] (1979 for EHV [82]). Thus, molecular dating efforts have relied on extant sequences, with the difficulties associated with the TDRP. Studies that did not account for the TDRP provided estimates of the time to the most recent common ancestor (TMRCA) of HCV genotypes in a range between ∼200 and 1000 years ago [64], [76], [87], [88], [63]; the origin of the horse virus was dated around 1800 CE [85]. A study that separately accounted for the rate of synonymous and nonsynonymous substitutions estimated HCV to have originated at least 2000 years ago [89]. Recently, a method based on an a selection-informed model was used to estimate the divergence time of HCV genotypes and the origin of extant EHV/CHV strains [70]. This approach, provided estimates of ∼3000 years ago for the TMRCA of extant HCV genotypes (with a low-bound estimate of ∼5000 years before present) and of ∼800 years ago for EHV/CHV [70]. If these dates are taken to provide at least an indication of the real evolutionary scenario, the possibility that HCV was transmitted to humans by horses infected with EHV can be excluded, an observation in line with the low diversity of EHV/CHV [82]. The origin of EHV/CHV as reverse zoonosis (i.e., the transmission of a human virus to animals) seems also unlikely, as in this case horse viruses should cluster within HCV diversity, unless the HCV lineage that originated EHV went extinct in the last 800 years. Indeed, as the HBV story exemplifies, viral lineage extinction can occur and was previously documented for other human pathogens such as parvovirus B19 [90], and variola virus [91]. As anticipated above, breeding practices may facilitate this process in the case of animal viruses. We know, for instance, that a minimum of two horse lineages went extinct during the domestication process and that horse genetic diversity has largely been shaped by events that occurred in the last few centuries [92]. It is thus possible that human-mediated selection on the host also resulted in the artificial selection of viral lineages. This would explain the relatively recent origin of extant EHV strains and their low diversity. If viral lineage extinction did occur, the time frames of EHV and HCV evolution would be underestimated and the scenario of HCV originating as a zoonosis from horses (or EHV as a reverse zoonosis) may still hold (Fig. 3C). Of course, the alternative possibility that EHV and HCV were transmitted independently to their present-day hosts by a third unknown reservoir is also in line with data on extant diversity and, if the transmission to horses occurred recently, does not require to postulate viral lineage extinction. Thus, a number of open questions remain on the origin of HCV. Hopefully, technological advances that allow sequencing of trace genetic material from ancient samples will provide information on viral strains hosted by humans and horses back in the past. At the same time, the extensive application of metagenomic approaches to different animal hosts across diverse geographic regions will expand our knowledge on hepacivirus diversity and eventually uncover the direct ancestor of HCV (if it still exists). Indeed, the possibility that the different HCV genotypes derived from independent cross-species transmission events [85] would imply that viruses related to HCV are relatively common in the wild, thus giving good chances to be recovered in large field surveys.

## 6 Hepatitis D virus

HDV is a defective virus incapable of autonomous propagation [93]. Its genome, a self complementary circular RNA of ∼1700 nucleotides, encodes a single protein (the HDV-encoded delta antigen) (Table 1). HDV requires HBV surface proteins, that are complexed with the delta antigen, to form transmissible virions [94]. Thus, HDV is usually considered a satellite of HBV, although recent data have shown that other enveloped viruses can promote HDV propagation, at least *in vitro* (e.g. HCV, dengue virus, vesicular stomatitis virus) [95].

Genetic heterogeneity among strains is quite high for HDV, which is thus classified in eight different genotypes (from 1 to 8), although a three major genogroup classification was recently proposed (group 1 for previous genotype 1, group 2 for genotypes 2 and genotypes from 4 to 8, and group 3 for previous genotype 3) [96] HDV genetic diversity is highest in Africa, suggesting that the defective virus emerged in and spread from this continent [40], [97]. The evolutionary origin of HDV is nonetheless unknown. HDV-like circular RNAs were only recently described in birds, reptiles, amphibians, fish and insects [98], [99], [100]. However, these HDV-like elements were not found to be associated with hepadnavirus infection, reinforcing the idea the HDV is not necessarily only transmitted in conjunction with HBV-related viruses, at least in these animals [100]. Current evidence suggests that HDV evolved from the human cellular transcriptome [101].

## 7 Hepatitis E virus

The first indications that a virus was responsible for waterborne, epidemic hepatitis came from studies of Asian outbreaks in the 1950–70s and, in analogy to HCV, the agent was referred to as “epidemic non-A, non-B hepatitis” [102], [103], [104]. Hepatitis E virus was eventually isolated and sequenced in the early 1990s [105], [106]. Since then, a number of HEV strains responsible for human infection were identified.

HEV is a positive-strand RNA virus belonging to the *Hepeviridae* family (Table 1). In common with all other members of this viral family, the HEV genome comprises three partially overlapping open reading frames (ORFs): ORF1 and ORF2 encode a non-structural polyprotein and the viral capsid, respectively, whereas ORF3 codes for a small phosphoprotein. A fourth ORF (ORF4), overlapping the helicase domain in ORF1, was recently described but seems to be specific for some HEV genotypes (HEV genotype 1, HEV-1) [107].

**Fig. 4:**
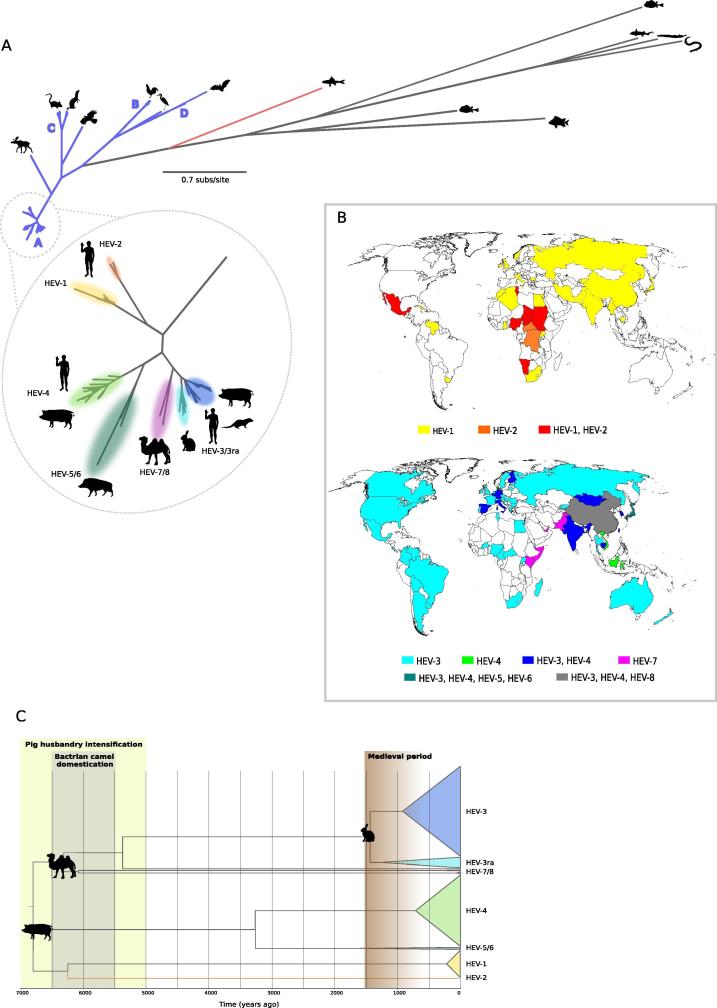
Hepevirus diversity and geographic distribution. (A) A maximum-likelihood phylogeny of the RdRp domain of known hepeviruses was generated with PhyML [136]. The Piscihepevirus branch is in red, Orthohepevirus branches are in blue. The enlargement shows phylogenetic relationships for viruses belonging to the *Orthohepevirus A* species, with representative hosts. (B) Geographic distribution of anthropotropic (HEV-1 and HEV-2) and enzootic (HEV-3–HEV-4) HEV strains. Genotypes were assigned to countries irrespective of their prevalence. Thus, even if a single case was reported in a given country, the genotype was recorded as present. Cases that could be clearly ascribed to migration/travels were excluded. Data derive from Forni et al. [121], with updates from [110], [140], [141], [142], [143], [144], [145], [146], [147], [148], [149], [150], [151]. (C) Time-scaled phylogenetic tree of a non-recombing ORF1 region [121]. Branch lengths represent evolutionary time. The time-frames of historical events mentioned in the text are reported. The rabbit and camel silhouettes mark the split of the rabbit-infecting and camel/dromedary-infecting genotypes. The pig silhouette marks the human-restricted/enzootic genotype split. (For interpretation of the references to colour in this figure legend, the reader is referred to the web version of this article.)

Members of the *Hepeviridae* family are currently classified into two genera, *Orthohepevirus* and *Piscihepevirus* [108] (https://talk.ictvonline.org/ictv-reports/ictv_online_report/positive-sense-rna-viruses/w/hepeviridae). The *Piscihepevirus* genus includes only one species (*Piscihepevirus A*) with one member (cutthroat trout virus), whereas the *Orthohepevirus* genus is divided into four species of viruses infecting mammals and birds (*Orthohepevirus A*–*D*) [108] (Fig. 4A). This classification is, however, likely to change in the near future following the identification of novel hepeviruses in fish other than trout and in amphibians [28] (Fig. 4A).

Human-infecting HEV strains are genetically heterogeneous and display distinct epidemiologic patterns, but all belong the *Orthohepevirus A* species. Orthohepeviruses A account for a minority of the overall diversity of hepeviruses that infect vertebrates and their closest relative is a presently unclassified virus detected in a Swedish moose (Fig. 4A) [109]. Field surveys revealed a high prevalence of HEV in moose populations from Sweden and other Baltic Regions [110], [111]. In general, ungulates represent major Orthohepeviruses A reservoirs. At present, eight Orthohepevirus A genotypes are recognized (HEV-1 to -8) (Fig. 4A). HEV-1 and HEV-2 infect only humans and cause waterborne outbreaks mainly in tropical and subtropical regions (Fig. 4B). Conversely, genotypes 3 and 4 account for the majority of hepatitis E human cases in industrialized countries. HEV-3 and HEV-4 also infect several other domestic (mainly pigs) and wild (e.g., ungulates and small carnivores) animals, their transmission to humans being usually zoonotic [2] (Fig. 4A). Phylogenetic analyses showed that HEV-3 and HEV-4 sequences derived from human cases are interspersed within those isolated from swine, indicating that pig-infecting HEV-3 and HEV-4 can easily cross the species barrier and infect humans [112]. Notably, though, evolutionary rates are higher for genotypes 3 and 4 than for the human-specific genotypes, suggesting cyclical adaptation to different mammalian hosts [113]. A distinct HEV-3 clade, mainly detected in rabbits (HEV-3ra), can also cause human hepatitis E [114], [115], [116], [117] (Fig. 4A). The remaining genotypes HEV-5/HEV-6 and HEV-7/HEV-8 have been detected in boars and camels, respectively [2] (Fig. 4A). However, they are also thought to have zoonotic potential, as HEV-5 and HEV-7 can infect cynomolgus monkeys [118], [119] and HEV-7 was detected in a patient who consumed camel meat and milk [120]. Thus, viruses belonging to all HEV genotypes seem to be transmissible to humans. Conversely, experimental infection with HEV-1 and HEV-2 indicated that these viruses have a host range restricted to primates [2]. HEV genotypes are therefore usually referred to as enzootic (HEV-3 and −4) or human-restricted/anthropotropic (HEV-1 and -2).

Although several human hepatitis E cases have a zoonotic origin and orthohepeviruses A are found in diverse mammals, recent data indicated that one or more reverse zoonoses led to the emergence and radiation of HEV genotypes [121]. In fact, character state reconstruction on a large phylogeny revealed that humans were the most likely hosts of the ancestor of extant orthohepeviruses A [121]. This notion is in line with the observation that most, if not all, HEV genotypes can infect our species, whereas other animals are differentially susceptible to distinct HEV genotypes. Moreover, increasing evidence suggests that reverse zoonotic events (also known as zooanthroponoses) are all but rare, and examples include other RNA viruses such as rotaviruses, enteroviruses, and human influenza viruses [122], [123], [124], [125]. For both swine influenza A viruses and swine vesicular disease virus onward transmission in pigs is well documented [123], [125] and is facilitated by intensive husbandry practices.

Molecular dating using a selection-informed method inferred that the ancestor of extant orthohepeviruses A existed ∼6800 to ∼3200 years ago, most likely in East Asia [121]. These inferences well correlate with historical circumstances that may have favored HEV emergence and host range expansion. In this period, sedentary agriculture promoted the appearance of large human settlements in several Asian regions and pig husbandry practices started to intensify in East Asia [126], [127], [128], [129], [130] (Fig. 4C). Crowded living conditions and poor sanitation possibly favored the emergence and spread of the waterborne, human-specific HEV strains. The close contact between humans and pigs most likely promoted HEV zooanthroponotic transmission and emergence of the enzootic strains (Fig. 4C). Additional reverse zoonotic transmissions may have also originated the camel-infecting and rabbit-infecting strains. In fact, the estimated timing of HEV-7/8 emergence (4055 BCE to 1192 BCE) [121] encompasses the time of domestication of Bactrian and dromedary camels [131], [132], [133] (Fig. 4C). As for HEV-3ra, it was estimated to have diverged from HEV-3 around 600 CE, in Europe [121]. This time frame corresponds to the Middle Ages, when historical evidence indicates that rabbits were kept in warrens and bred for meat [134] (Fig. 4C). Of course, these estimates do not necessarily imply that camels and rabbits acquired HEV from humans, as the domestication process may have exposed these animals to various viral sources, including other domestic (e.g., pigs) and peridomestic mammals.

These scenarios provide a credible framework for orthohepevirus A origin, as well as for the diversification of HEV genotypes, and selection-informed methods should at least partially correct for the TDRP, as they explicitly model purifying selection [14], [15], [16]. Nonetheless, the above-mentioned data on HBV [9] suggest caution in the inference of time and location of ancestral events based on extant viral diversity. Also, pig infection with HEV-3 and HEV-4 is generally asymptomatic [135], possibly indicating a long-standing host-virus association that might even predate swine husbandry development.

It should also be noted that the ultimate origin of orthohepevirus A remains unknown. Humans may have acquired HEV through cross-species transmission from other animals. However, known orthohepeviruses that infect mammals and birds are distantly related to orthohepevirus A, indicating that none of them represents the source of human-infecting HEV. Likewise, the origin and evolutionary relationship between the moose virus and human-infecting orthohepeviruses remain unclear.

## 8 Conclusions

By allowing the large-scale identification of novel viral species, as well as the sequencing of viral genomes from archaeological samples, technological advances have largely expanded our knowledge on the evolution and origin of human hepatitis viruses. These insights have been paralleled by the development of computational tools and theoretical frameworks to analyze and mine viral sequence data (e.g., the above-mentioned recognition of the TDRP and the development of approaches to correct for it). The overall picture emerging from these studies clearly indicates that, with the possible exception of HDV, viruses related to human hepatitis viruses infect several other mammalian and non mammalian vertebrates. The specific events that originated these human pathogens remain, however, largely unknown. For HCV and HEV, the evolutionary history of the human viruses is likely intertwined with that of related viruses that infect domestic animals. Conversely, in the case of HAV and HBV, the closest relatives of the human viruses are found in NHPs. In general, these observations indicate that a deeper understanding of the evolutionary dynamics of human viruses has a relevance not only to improve our ability to treat and prevent present infections, but also to gain insight into the ecological contexts that may fuel the emergence of novel human pathogens, with particular reference to zoonotic ones.

## Declaration of Competing Interest

The authors declare that they have no known competing financial interests or personal relationships that could have appeared to influence the work reported in this paper.
